# Decrease in electrolyte after vitrectomy surgery may affect the results of forensic investigations using vitreous humor

**DOI:** 10.1186/s12886-024-03445-2

**Published:** 2024-05-23

**Authors:** Hiroaki Ushida, Ayana Suzumura, Kazuhisa Yamada, Hideyuki Shimizu, Atsuo Suzuki, Yusuke Ishikawa, Ryosuke Kikuchi, Koji M. Nishiguchi, Hiroki Kaneko

**Affiliations:** 1 Department of Ophthalmology, Nagoya University Graduate School of Medicine, 65 Tsurumai-cho, Showa-ku, 466-8550 Nagoya, Japan; 2 Department of Clinical Laboratory, Nagoya University Hospital, 65 Tsurumai-cho, Showa-ku, 466-8550 Nagoya, Japan; 3 Division of Clinical Laboratory, Gifu University Hospital, 1-1 Yanagido, 501-1194 Gifu City, Japan

**Keywords:** Vitrectomy surgery, Intravitreal electrolytes, Cataract surgery, Forensic investigation, Postmortem interval

## Abstract

**Purpose:**

Vitreous humor (VH) is used for postmortem biochemical studies because it is well protected in an uncontaminated state even after death. The goal of this research was to investigate electrolyte concentrations in the VH from human eyes with and without a history of vitrectomy surgery.

**Methods:**

We analyzed the sodium (Na), potassium (K), chloride (Cl) and magnesium (Mg) concentrations from 34 VH samples from 34 patients. Eleven samples were from eyes with a history of vitrectomy, and the remaining 23 eyes had no history of vitrectomy. The correlations of Na, K, Cl and Mg concentrations with patient age, interval between first and second vitrectomy, and lens status (history of cataract surgery) were also evaluated.

**Results:**

The Na, K, Cl and Mg concentrations in VH from vitrectomized eyes were 134.1 ± 7.9 mmol/L, 3.7 ± 0.2 mmol/L, 99.7 ± 6.7 mmol/L and 0.59 ± 0.09 mmol/L, respectively; all were significantly lower than the corresponding concentrations in VH from control eyes (lower by 5.0%, 11.0%, 11.7%, and 22.6%, respectively). Na, K, Cl and Mg concentrations in VH from vitrectomized eyes did not show significant correlations with patient ages or the interval between their first and second vitrectomies. There were no significant differences in Na, K, Cl and Mg concentrations in VH between phakic eyes and intraocular lens-implanted eyes.

**Conclusions:**

With the increasing number of vitrectomies being performed, it is necessary to consider the history of vitrectomy when using a subject’s VH in forensic examination.

**Supplementary Information:**

The online version contains supplementary material available at 10.1186/s12886-024-03445-2.

## Background

Measuring postmortem time is critical in medical and legal investigations, and doing so accurately is a challenge for forensic pathologists. In particular, accurate estimation of the postmortem interval (PMI), defined as the amount of time that has elapsed since the death of the decedent [1], is a useful statistic for establishing timeframes for crimes, narrowing the list of suspects during police investigations of crimes with no witnesses, and resolving civil cases such as property inheritance and insurance-policy disputes [2, 3]. Closed-compartment body fluids are well protected and not immediately contaminated postmortem, thus, the focus has shifted to biochemical assessment of various parameters to accurately estimate PMI [4, 5]. Of note, it has been reported that, even shortly after death, the vitreous humor (VH) is largely uncontaminated. Therefore, VH is suitable for use in postmortem biochemical investigations [6]. Previous studies indicate that increasing potassium (K), decreasing sodium (Na), and decreasing chloride (Cl) all have strong correlations with increasing PMI, and that increasing magnesium (Mg) has a slight correlation with PMI [7].

With the increase in life expectancy, the number of ophthalmic diseases and ophthalmic surgeries is increasing. As cataracts remain, by far, the number one cause of blindness in the world [8], cataract surgery is currently one of the most commonly performed procedures in developed countries. Indications for cataract surgery are changing, with more patients being operated on at a younger age and with superior vision [9]. The number of surgeries per year is increasing and is expected to double within the next 20 years [10, 11]. Vitrectomy is one of the most-common ophthalmic surgical procedures for many retinal diseases, e.g., retinal detachment, diabetic retinopathy and macular hole [12–19]. It is estimated that over 500,000 people worldwide undergo a vitrectomy each year [20]. Similar to cataract surgeries, vitrectomy rates have increased in recent decades [21].

Liquid VH is about six times more viscous than saline [22], but vitrectomies remove the vitreous. Therefore, there could be many differences between eyes with and without a history of vitrectomy. For example, there are reports that the duration of effect of anti-vascular endothelial growth factor drugs differs between eyes with and without a history of vitrectomy [23], and that inflammatory cytokine levels in the aqueous humor are significantly higher in eyes with diabetic macular edema that have undergone vitrectomy than in those that have not [24]. It is highly likely that a vitrectomy may alter not only cytokines and fluid flow, but also other factors, including electrolytes, in the VH that is used for the measurement of PMI. However, to the best of our knowledge, no single paper analyzing VH after vitrectomy has been reported. Therefore, in this study, we collected VH from eyes that had a history of vitrectomy more than six months previously and were scheduled to undergo vitrectomy again and examined the electrolytes in the VH.

## Materials and methods

### Sample collection and patient characteristic

This study was conducted in the Nagoya University Hospital between September 2020 and June 2022. Sample collection was described previously [12, 25]. Briefly, we collected VH from the eyes of patients with the history of previous vitrectomy (Vitrectomized VH), and from eyes of patients without a history of vitrectomy (Control VH) as the control group. For the surgeries and VH collection, we used a 25-gauge three-port vitrectomy system with the Constellation Vision System (Alcon Laboratories, Inc, Fort Worth, TX, USA) with a vitrectomy cutter and aspirated VH before starting infusion to prevent contamination of balanced salt solution. No data were missing. Data were fully anonymized before analysis. This study was conducted in accordance with the guidelines of the Declaration of Helsinki; the protocol was registered within the UMIN Clinical Trial Registry (registered number UMIN000024553) and the Nagoya University Hospital Ethics Review Board approved the protocol (2013-0010, 2020 ‐ 0598). We obtained written informed consent from all participating patients. All samples were centrifuged, and we used only the supernatants. We stored the samples at − 80 °C until use.

### Patient characteristics

The characteristics of the study patients are shown in Table 1.

**Table 1:** Patient characteristics

No. of patients (number of men)		Age (years)	Retinal disease requiring vitrectomy
History of vitrectomy surgery			Primary (number)
+ (Vitrectomized VH)	11(7)	61.5 ± 12.2	MH (4)
			RD (3)
			IOL drop (2)
			Aphakia (2)
− (Control VH)	23(15)	64.7 ± 12.7	MH (10)
			VMT (3)
			ERM (6)
			RD (3)
			IOL dislocation (1)

MH: macular hole, RD: retinal detachment, IOL: intraocular lens, VMT: vitreomacular traction, ERM: epiretinal membrane

Data are shown as mean ± standard deviation

We analyzed VH samples from one eye in each of the 34 patients. Of the 34 eyes, 11 had a history of vitrectomy (Vitrectomized VH) and the other 23 had no history of vitrectomy (Control VH).

### Laboratory investigations

We measured concentrations of electrolytes (Na, K, Cl and Mg) in the VH samples using a LABOSPECT 008 (Hitachi High-Technologies Corporation, Tokyo, Japan) and compared the average values between groups.

### Statistics

We express the data as means ± standard deviation (n = number of samples). For patients who had both eyes operated on at different times for different reasons, each eye was counted separately ( n = 2). We used the Mann-Whitney U test. We analyzed the possible correlations between patients’ ages, years between first and second vitrectomies and electrolyte levels using Spearman’s rank correlation. P-values < 0.05 were considered statistically significant in all analyses.

## Results

### The effect of vitrectomies on the VH electrolytes

**Fig. 1:**
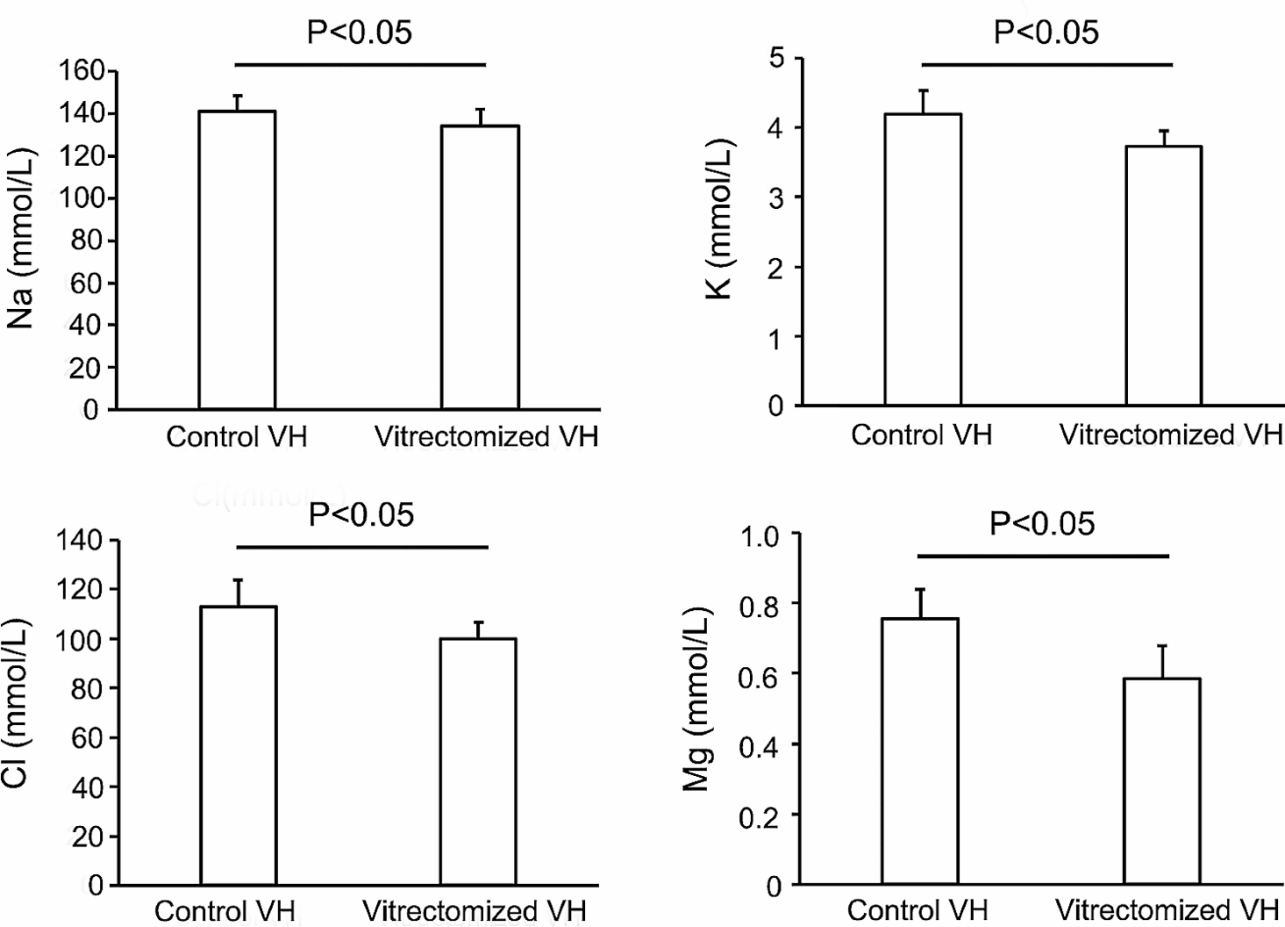
**Comparison of vitreous electrolytes concentrations between the eyes with and without a history of vitrectomy.** Na, K, Cl and Mg concentrations in vitreous humor (VH) from eyes with the history of vitrectomy (Vitrectomized VH) are compared with those from eyes with no previous vitrectomy (Control VH). Na, K, Cl and Mg concentrations in Vitrectomized VH were significantly smaller than those in Control VH. See text for p-values. Data are shown as mean ± standard deviation.

The 11 Vitrectomized VH patients (including seven males) had a mean age of 61.5 ± 12.2 years. The causes necessitating the second vitrectomy were macular hole in four eyes, rhegmatogenous retinal detachment in three eyes, intraocular lens (IOL) drop in two eyes and artificial aphakia in two eyes. The 23 Control VH patients (including 15 males) had a mean age of 64.7 ± 12.7. The Control VH eyes included 10 eyes with a macular hole, three with vitreomacular traction, six with an epiretinal membrane, three with rhegmatogenous retinal detachment and one eye with a dislocated IOL. The concentrations of Na, K, Cl and Mg in Vitrectomized VH eyes were 134.1 ± 7.9 mmol/L, 3.7 ± 0.2 mmol/L, 99.7 ± 6.7 mmol/L and 0.59 ± 0.09 mmol/L, respectively. In Control VH eyes, the Na, K, Cl and Mg concentrations were 141.3 ± 7.3 mmol/L, 4.2 ± 0.3 mmol/L, 113.0 ± 10.6 mmol/L, and 0.76 ± 0.08 mmol/L, respectively. The measured electrolytes were significantly lower in Vitrectomized VH eyes than in Control VH eyes, by 5.0% for Na, 11.0% for K, 11.7% for Cl and 22.6% for Mg (Fig. 1).

### The relationship between the patients’ ages and the VH electrolytes

**Fig. 2:**
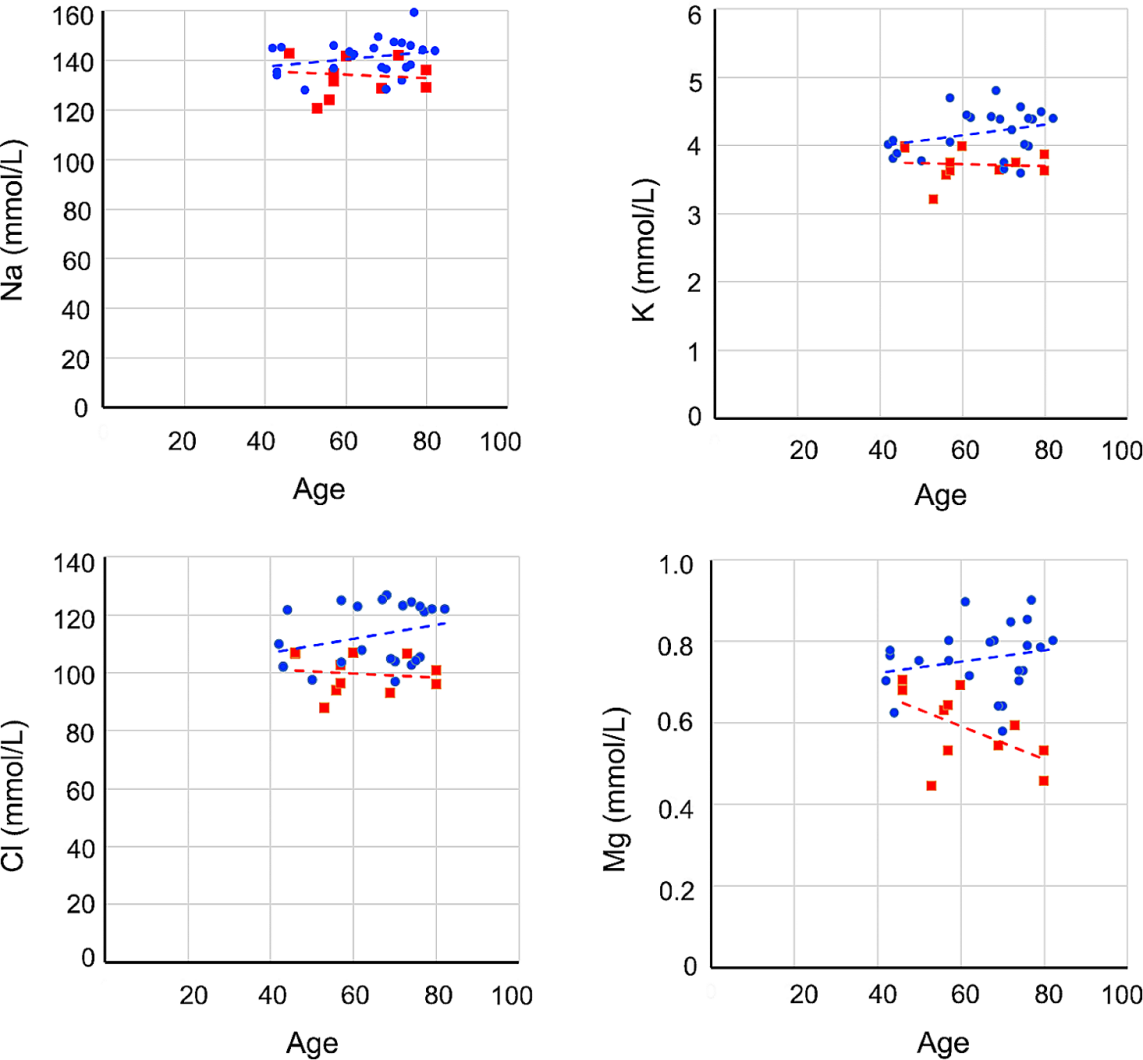
**Correlation between vitreous humor (VH) electrolyte concentrations and patient age in eyes with and without previous vitrectomy surgery.** Red dots and lines indicate electrolyte concentrations in VH from eyes with a history of vitrectomy (Vitrectomized VH). Blue dots and lines indicate electrolyte concentrations in VH from eyes without a history of vitrectomy (Control VH). Although no significant correlations were observed for the Na, K, Cl and Mg concentrations, trends toward increasing concentrations with increasing age were observed in Control VH. Conversely, although no the correlations were not significant for Na, K, Cl and Mg concentrations, trends toward decreasing concentrations with increasing age were observed in Vitrectomized VH.

We next investigated whether electrolyte concentrations were correlated with the patients’ ages (Fig. 2). In the Vitrectomized VH group, the concentrations of Na (*P* = 0.66, ρ = −0.151), K (*P* = 0.83, ρ = −0.074), Cl (*P* = 0.68, ρ = −0.143) and Mg (*P* = 0.16 ρ = −0.459) in the VH tended to decrease with age but did not show any significant correlation with age. In the Control VH group, the concentrations of Na (*P* = 0.23, ρ = 0.259), K (*P* = 0.46, ρ = 0.161), Cl (*P* = 0.27, ρ = 0.238) and Mg (*P* = 0.23, ρ = 0.174) showed a trend of concentrations to increase with age but, again, did not show a significant correlation with age.

### The effect of vitrectomy on the VH electrolytes

As the next step in our study, we determined whether the interval between the first and second vitrectomies affected the concentrations of VH electrolytes. Table 2 presents the characteristics of the patients (eyes) that had a second vitrectomy.

**Table 2:** The characteristics of the eye with the hisotry of previous vitrectomy

Age range	Sex	R/L	Disease for the 1st vitrectomy	Disease for the 2nd vitrectomy	Interval between 1st and 2nd vitrectomy
55–65	M	R	ERM	MH	6 m
75–85	M	R	RD	IOL drop	20y
75–85	M	R	MHRD	RD	1.5y
45–55	F	L	PDR	MH	7y
55–65	M	R	ERM	RD	2y
55–65	F	L	RD	IOL drop	9y
45–55	M	L	RD	aphakia	20y
45–55	M	R	RD	aphakia	18y
65–75	F	L	MH	MH	6 m
55–65	F	R	MH	MH	1y
65–75	M	L	RD	RD	1y

MH: macular hole, RD: retinal detachment, IOL: intraocular lens, VMT: vitreomacular traction, ERM: epiretinal membrane, MHRD: macur hole-associated retinal detachment, PDR: proliferative diabetic retinopathy

The time between the first and second surgeries ranged from about 6 months up to 20 years.

**Fig. 3:**
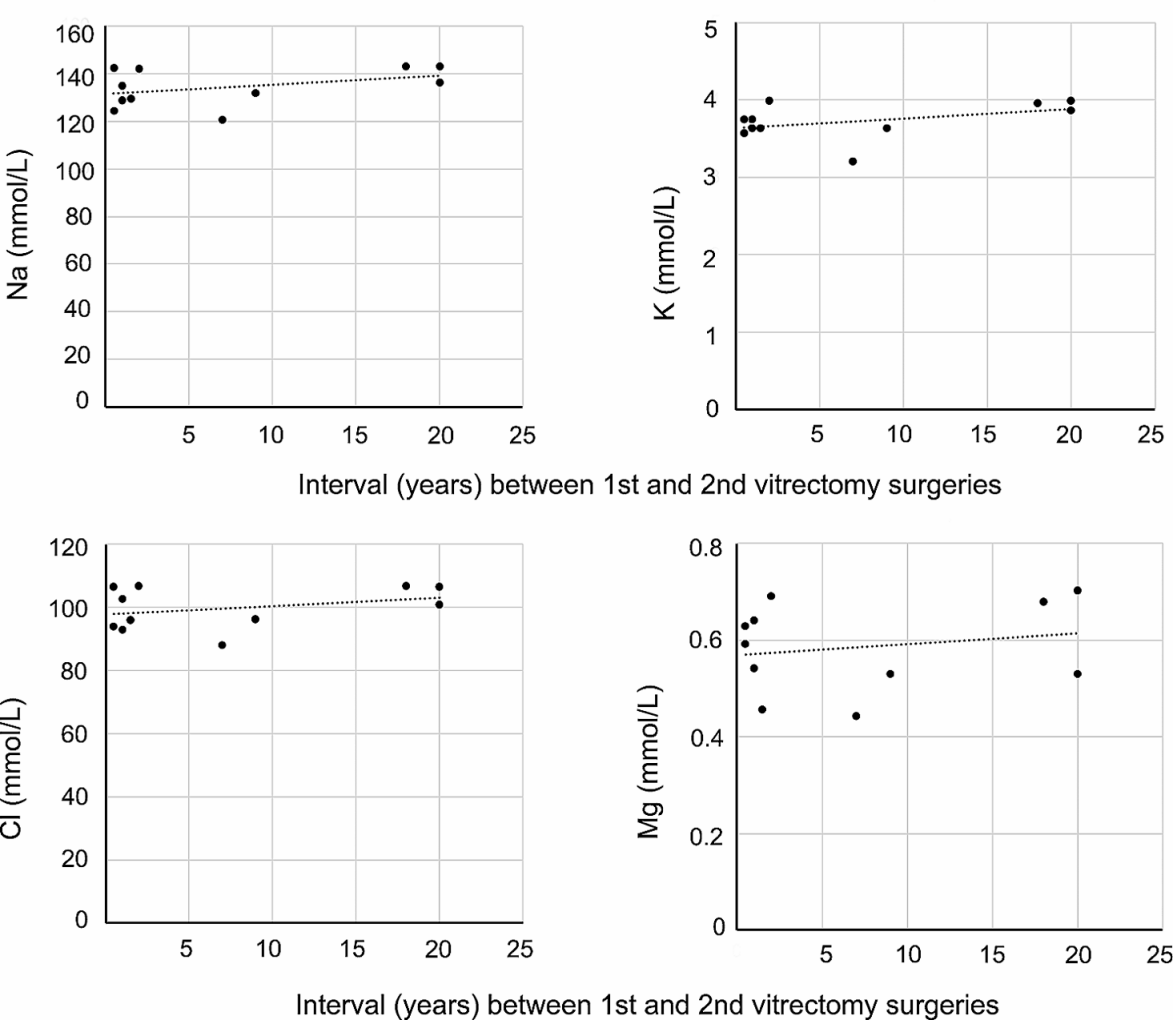
**Correlation of electrolyte concentrations with the years between first and second vitrectomies.** There were no significant correlations between Na, K, Cl and Mg concentrations and the interval since the first vitrectomy, but some trends toward increasing concentrations with increasing years between first and second vitrectomy surgery were observed.

We investigated whether electrolyte concentrations in VH of eyes in the Vitrectomized VH group were associated with the length of the interval between the patients’ first and second vitrectomies (Fig. 3). Although there was a tendency for electrolyte concentrations to increase with longer intervals before the second surgery, Na (*P* = 0.20, ρ = 0.415), K (*P* = 0.18, ρ = 0.440), Cl (*P* = 0.42, ρ = 0.271) and Mg (*P* = 0.85, ρ = 0.067) concentrations were not correlated with the interval between the two surgeries.

### The effect of lens status on the VH electrolytes

**Fig. 4:**
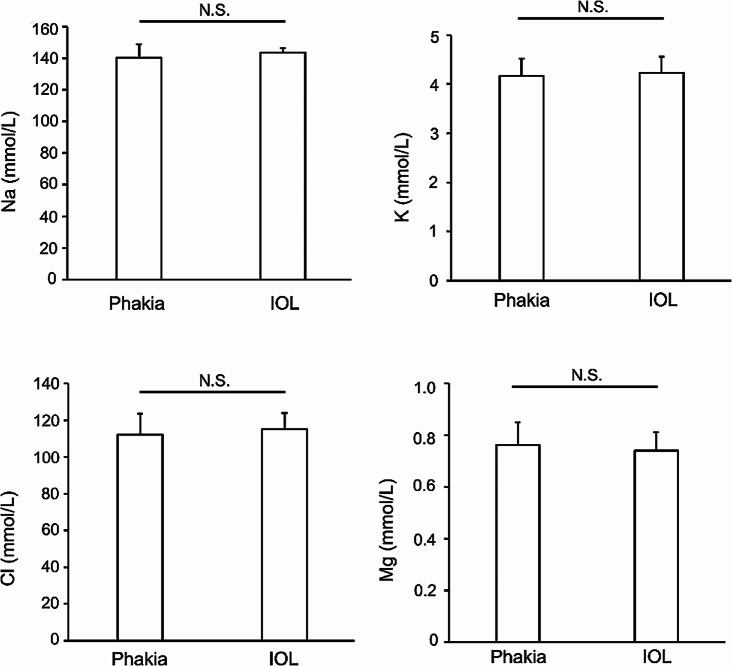
**Comparison of vitreous electrolytes concentrations in different lens conditions.** Na, K, Cl and Mg concentrations in vitreous humor from eyes without prior vitrectomy surgery were divided into two groups, those with no history of cataract surgery (Phakia) and those with a history of cataract surgery (IOL: intraocular lens). There were no significant differences between groups (*P* > 0.05) in Na, K, Cl and Mg. Data are shown as mean ± standard deviation.

Finally, we examined the possible correlation between a history of cataract surgery and the electrolytes (Fig. 4). Na, K, Cl and Mg concentrations in the Control VH group were divided into two groups: those without a history of cataract surgery (Phakia: 17 eyes) and those with a history of cataract surgery (IOL: six eyes) to determine whether the lens condition affected electrolytes. Those without previous cataract surgery had Na, K, Cl and Mg concentrations of 140.5 ± 8.3 mmol/L, 4.2 ± 0.4 mmol/L, 112.1 ± 11.4 mmol/L and 0.76 ± 0.09 mmol/L, respectively. In those with previous cataract surgery, Na, K, Cl, and Mg concentrations were 143.5 ± 2.8 mmol/L, 4.2 ± 0.3 mmol/L, 115.3 ± 8.5 mmol/L, and 0.74 ± 0.09 mmol/L, respectively. There were no significant differences (*P* > 0.38) in electrolytes Na, K, Cl and Mg based on lens status.

### Multivariate analysis

Multivariate analysis was performed using the electrolyte concentrations as the objective variable and age, history of vitrectomy, lens condition, and surgical interval as the explanatory variables. The last sample lacked axial length data and was excluded from the multivariate analysis. The results showed an association between K concentration and history of vitrectomy, with the K concentration being decreased with a history of vitrectomy (*P* < 0.05). However, K concentration was not associated with age, lens condition, surgical interval, or axial length. Meanwhile, Na, Cl, and Mg concentrations were not associated with age, lens condition, surgical interval, history of vitrectomy, or axial length.

## Discussion

VH has been extensively researched for use in postmortem biochemical investigations for the purpose of postmortem diagnosis of pre-existing diseases and elucidation of forensic questions, especially for determination of PMI [26]. In this study, we examined the influence of a history of major ophthalmic surgeries, such as cataract and vitreous surgeries, which have been increasing in number recently, on the results of forensic examinations. Our results showed that intravitreal Na, K, Cl and Mg were significantly lower in the VH of eyes with a history of vitrectomy than in those without such history. Regarding the size of the differences relative to Control VH levels, Vitrectomized VH eyes showed 5% less Na, 11% less K, 12% less Cl and 22% less Mg. Although the Mg concentration in the VH of the control group of living humans in our study was slightly lower than previously reported [27], the Mg concentration in the VH after vitrectomy was even lower than those values.

Mg is the fourth-most-abundant cation in the body and the second-most-abundant cation in intracellular fluid. In the human body, Mg exists in three states: ionized (free) and physiologically active, in complexes with anions, and bound to proteins. Most of the Mg in the human body is present in bone, soft tissue and muscle, with less than 1% present in blood [28]. Farmer et al. discussed postmortem Mg behavior, asserting that Mg is less useful than K in determining the PMI because environmental conditions such as site temperature, airflow and humidity (e.g., salt-water immersion deaths and fire deaths) affect Mg concentration in the vitreous [7].

Before death, K concentrations are reportedly low in the VH and high in the peripheral tissues of the eye, including the choroid, and retina [29, 30]. After death, reversal of the K gradient in the VH proceeds, and elevation of K in the VH occurs in a time series. A previous comparative study on synovial fluid and VH concluded that the K concentrations in both samples have a relationship with the PMI, but the synovial fluid K concentration has a stronger relationship with the PMI than the VH K concentration [31]. Another study compared VH and cerebrospinal fluid and concluded that VH was more suitable and stable than cerebrospinal fluid for estimating PMI [32]. In our study, we found that there was significant 11% reduction in K concentration in the VH in vitrectomized eyes relative to non-vitrectomized eyes, so a history of vitrectomy may impact the assessment of PMI resulting from the measurement of VH K concentration.

The limitations of this study are as follow: (1) It is not fully known why these four electrolytes decline after vitrectomy and what role they play in the VH. For example, Mg, the most reduced of the four electrolytes, is not charged in the body, therefore most Mg binds to proteins (mainly albumin) and circulates. It is reasonable to think that Mg and albumin change in parallel. Reportedly, VH from eyes with retinal disease had approximately three times the normal albumin concentration [33]. We also attempted to measure albumin concentrations in VH in this study but, unfortunately, albumin levels could not be measured in many Vitrectomized VH and some Control VH samples (below the detection sensitivity), making it impossible to statistically evaluate or correlate albumin with electrolytes. (2) The number of VH samples from eyes with a history of vitrectomy was low: in this study 11 VH samples were obtained, 2 of which were from the same patient (left and right eyes from different times). This number reflects the number of eyes that underwent a vitrectomy surgery at least twice. Although the number of vitrectomies is increasing worldwide, the number of cases in which two vitrectomies are performed on the same eye is quite limited. (3) It was not possible to investigate the differences in electrolytes for all the various retinal diseases for which a vitrectomy is indicated. We cannot exclude the possibility that the electrolytes in the vitreous may differ in different retinal diseases, e.g., diabetic retinopathy and retinal detachment. (4) The correlations between electrolyte concentrations in the blood and VH are still not fully understood. Because blood electrolyte measurements are not part of the routine preoperative blood testing, our study could not investigate the relationship between preoperative blood and VH electrolyte concentrations. (5) Previous reports have been highly controversial due to the large differences in results obtained between the right and left eyes of patients [34, 35]. However, due to the limited number of samples, there were insufficient numbers for direct comparisons of electrolyte concentrations in the right and left eyes of the same patients. (6) Although there was an interval of at least 6 months between 1st and 2nd vitrectomy surgeries in Vitrectomized VH, we cannot fully exclude the possibility that inflammation after the first surgery or other factors affected the current results. (7) There have been many reports in the literature regarding use of the K concentration in the VH for PMI determination. However, the K concentration in the VH is rarely used on its own to determine PMI. Attempts to use the rate of increase in VH K concentration for PMI determination cannot currently be considered standard because of the possibility of variations in values [36]. In Germany, this method has never been applied to forensic case processing, but there have been cases in which the PMI was identified based on the VH K concentration and the results of immunohistological staining [37]. Many studies have been conducted and some questions have been raised regarding the accuracy of estimating time of death based on increases in the K concentration. This is because the correlation between K concentration and PMI depends on a variety of factors, including cause of death, ambient temperature, duration of distress, antemortem electrolyte dysregulation, and pre-existing K levels [38]. In addition to PMI estimation, Na, K, Cl, and Mg concentrations are used as necrochemical markers of electrolyte imbalance, dehydration, exogenous salt intoxication, and drowning [39, 40]. However, as mentioned above, the Vitrectomized VH group in the present study had a small sample size, making it statistically unrealistic to subdivide the group by background retinal disease. (8) A report has pointed out that the preanalytical VH handling and analytical methods are important factors for the accuracy of K measurements and, by extension, the accuracy of PMI estimation. A report indicating high variability may reflect errors introduced by sample manipulation prior to analysis [41]. Although we carefully handled samples immediately after the surgeries, there is a possibility that handling in the surgical condition might affect the results. (9) In eyes with a long axial length, the volume is large and liquefaction can progress, which may affect the electrolyte concentrations. Therefore, in this study, we investigated whether significant differences existed in age and axial length between Vitrectomized VH and the Control VH group. The results revealed that there was no significant difference in age between the two groups. However, a significant difference (*P* < 0.01) in ocular axis length was observed, with the ocular axis being longer in Vitrectomized VH group. Another report on cataract surgery cases indicated that many cytokines did not show changes dependent on the ocular axial length [42]. However, we cannot exclude the possibility that longer axial length might affect the results in this study.

## Conclusions

Intravitreal electrolyte concentrations were found to be lower in post-vitrectomized eyes than in control eyes. It is suggested that when using VH for autopsies, it is necessary to check the subject’s history of ophthalmic surgery. Interestingly, to the best of our knowledge, there have been no previous studies investigating VH in eyes with a history of vitrectomy. With the increase in the number of such surgeries in recent years, it may gradually become more difficult to use VH as the basis for conventional autopsy decisions without taking past vitrectomies into account. It is hoped that a larger number of studies will be conducted on VH in eyes with a history of vitrectomy, and that the characteristics of this VH will be clarified.

### Electronic supplementary material

Below is the link to the electronic supplementary material.

Supplementary Material 1

## Data Availability

All data generated or analysed during this study are included in this published article and its supplementary information files.
